# Diastolic echocardiographic parameters identify pediatric kidney transplant recipients with structural myocardial alterations

**DOI:** 10.1007/s00467-026-07416-1

**Published:** 2026-06-27

**Authors:** Jeannine von der Born, Tim Alexander Ubenauf, Rizky I. Sugianto, Carl Grabitz, Elena Lehmann, Nima Memaran, Nigar Babazade, Samir Sarikouch, Diane Miriam Renz, Bernhard Magnus Wilhelm Schmidt, Anette Melk

**Affiliations:** 1 Department of Pediatric Kidney- and Liver Diseases, Metabolics and Neuropediatrics, Hannover Medical School, Carl-Neuberg-Str. 1, 30625 Hannover, Germany; 2 Hannover Medical School, AVIATOR – Advanced Clinician Scientist Program, Dean’s Office for Academic Career Development, Carl-Neuberg-Str. 1, 30625 Hannover, Germany; 3 Hannover Medical School, KlinStrucMed Program, Dean’s Office for Academic Career Development, Carl-Neuberg-Str. 1, 30625 Hannover, Germany; 4 Institute of Diagnostic and Interventional Radiology, Hannover Medical School, Carl-Neuberg-Str. 1, 30625 Hannover, Germany; 5 Department of Cardiothoracic, Transplantation and Vascular Surgery, Carl-Neuberg-Str. 1, 30625 Hannover, Germany; 6 Department of Nephrology and Hypertension, Hannover Medical School, Carl-Neuberg-Str. 1, 30625 Hannover, Germany

**Keywords:** Children, Kidney transplantation, Diastolic dysfunction, Echocardiography, Native T1 time, Cardiac MRI

## Abstract

**Background:**

Cardiac complications are among the most common causes of death in patients after pediatric kidney transplantation (KTx), but defined diagnostic procedures identifying young patients at risk are not established. Cardiovascular magnetic resonance (CMR) imaging with native T1 mapping allows detection of diffuse myocardial alterations but is not routinely available for cardiovascular screening. Whether abnormalities detected by echocardiography reflect underlying myocardial structural changes remains unclear.

**Methods:**

Pediatric KTx recipients underwent comprehensive transthoracic echocardiography and CMR imaging with native T1 mapping. Associations between echocardiographic measures and T1 values were analyzed using multivariable linear regressions. Receiver operating characteristics analyses assessed the ability of septal E/e′ to identify elevated T1 values, with area under the curve (AUC) and optimal cut-offs determined using positive likelihood ratios (LR +).

**Results:**

Forty-six pediatric KTx recipients (16 ± 3.5 years old; time since KTx 7.9 ± 5.3 years) were included. Diastolic echocardiographic abnormalities were common, with 87% exhibiting at least one abnormal diastolic parameter. Septal T1 was associated with septal E/e′, A-wave, and pulmonary venous atrial reversal, while lateral T1 was associated only with septal E/e′. Optimal septal E/e′ cut-offs were 10.550 for detecting an elevated septal T1 (LR +  = 7.143) and 10.630 for detecting an elevated lateral T1 (LR +  = 9).

**Conclusions:**

Pediatric KTx recipients with structural myocardial alterations on CMR imaging exhibit detectable abnormalities in routine echocardiographic diastolic parameters. Especially a markedly elevated septal E/e′ could identify patients at increased risk for underlying myocardial involvement and justify the use of CMR imaging in post-transplant follow-up.

**Graphical abstract:**

**Figure:**
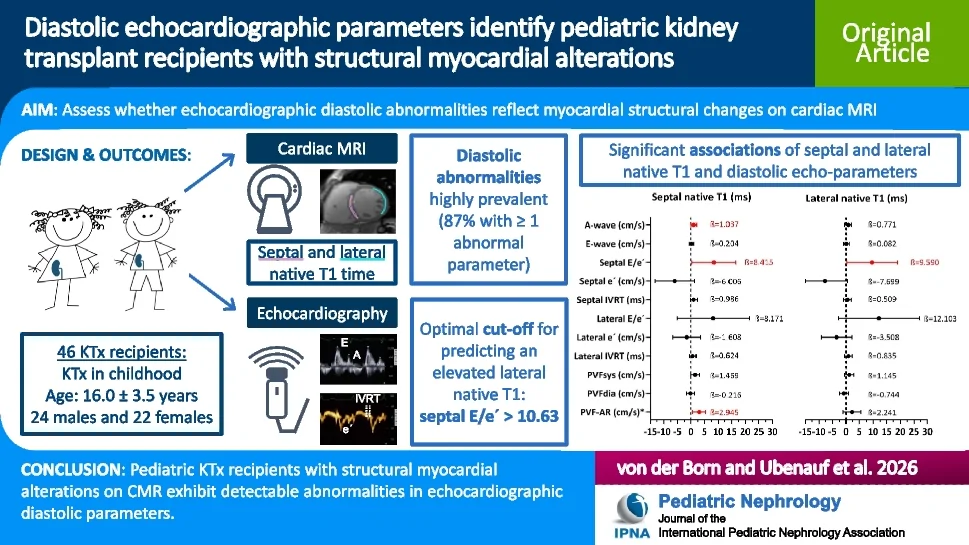
A higher resolution version of the Graphical abstract is available as Supplementary information

**Supplementary Information:**

The online version contains supplementary material available at https://doi.org/10.1007/s00467-026-07416-1.

## Introduction

Despite major advances in pediatric kidney transplantation (KTx), patient survival remains substantially reduced compared with the general population [1]. Cardiovascular disease represents a leading cause of morbidity and mortality in children and young adults after KTx, accounting for a considerable proportion of premature deaths already in adolescence and early adulthood [1–3]. Structural and functional cardiac alterations develop early in the course of chronic kidney disease (CKD), progress with declining kidney function, and frequently persist even after successful transplantation [4, 5]. Identifying children and adolescents at increased cardiovascular risk after KTx therefore remains a major clinical challenge in long-term post-transplant care.

Cardiovascular magnetic resonance (CMR) imaging has emerged as a powerful tool for the non-invasive assessment of myocardial structure. In particular, native T1 mapping allows the detection of diffuse myocardial fibrosis without the need for contrast agents [6]. Elevated native T1 time (T1) values have been associated with adverse cardiovascular outcomes and increased mortality in adult patients with advanced CKD and on dialysis [7, 8]. Myocardial fibrosis assessed by native T1 has also been described in KTx recipients, suggesting persistent structural myocardial damage despite restoration of kidney function [9, 10]. In a previous study conducted in the same pediatric KTx cohort, we demonstrated significantly elevated septal native T1 values compared with healthy controls, indicating diffuse myocardial alterations consistent with fibrosis [11]. However, routine implementation of CMR imaging in pediatric follow-up care is limited by restricted availability, long examination times, costs, and patient-related factors such as the need for cooperation during breath-hold sequences [12]. Consequently, CMR imaging cannot be applied as a universal screening tool in all pediatric KTx recipients. At present, there are no established strategies to identify those children who are most likely to benefit from advanced cardiac imaging, highlighting a relevant gap in post-transplant cardiovascular surveillance.

Echocardiography is widely available and routinely used in the follow-up of pediatric patients with CKD and after KTx. While systolic left ventricular function is typically preserved or recovers after transplantation, multiple studies have demonstrated that functional alterations predominantly affect diastolic parameters [13, 14]. Diastolic dysfunction has been shown to occur early in CKD, often independently of left ventricular hypertrophy, and to persist after transplantation [15, 16]. In broader pediatric and adult populations, diastolic dysfunction represents an independent predictor of adverse outcomes [17]. These findings suggest that diastolic echocardiographic parameters may serve as sensitive functional markers of early myocardial involvement in this high-risk population. Of particular interest is the septal E/e′ ratio, as it is well established in routine clinical practice, is easy and reliable to measure, and represents a key parameter in the noninvasive assessment of left ventricular diastolic function, providing an accurate estimate of left ventricular end-diastolic filling pressures [18, 19].

Despite growing evidence for both structural myocardial alterations detected by CMR imaging and functional abnormalities assessed by echocardiography in pediatric KTx recipients, data linking these two modalities are scarce. In particular, it remains unclear whether echocardiographic diastolic abnormalities already reflect underlying structural myocardial damage, such as diffuse fibrosis detected by native T1 mapping. Establishing such an association would be of considerable clinical relevance, as it could enable the use of echocardiography as a pragmatic first-line tool to identify patients at increased risk for myocardial injury and to guide the targeted use of CMR imaging.

Therefore, the aim of this study was to comprehensively characterize echocardiographic diastolic function in children, adolescents, and young adults after pediatric KTx and to investigate the association between diastolic echocardiographic parameters and structural myocardial alterations assessed by native T1 mapping on CMR imaging. By linking routine echocardiographic findings with advanced imaging markers of myocardial damage, we sought to identify clinically applicable parameters that may support cardiovascular risk stratification in long-term post-transplant follow-up.

## Methods

### Study population

For this monocentric, cross-sectional study, KTx recipients were recruited from the outpatient clinic of the Department of Pediatric Kidney- and Liver Diseases, Metabolics and Neuropediatrics at Hannover Medical School (Hannover, Germany). Eligible participants had received KTx during childhood at least 6 months prior to inclusion and were required to be capable of undergoing a sedation-free CMR examination, including breath-hold sequences. Exclusion criteria comprised current dialysis treatment, acute graft rejection, age younger than 7 years or older than 25 years, and any contraindication to CMR imaging.

Clinical data including underlying kidney disease, transplantation characteristics, cumulative duration of dialysis prior to transplantation, current medication, and laboratory parameters were obtained from medical records. Estimated glomerular filtration rate (eGFR) was calculated using the Schwartz formula (0.41 × height [cm]/serum creatinine [mg/dL]) [20].

For CMR analyses, results from the KTx cohort were compared with those from an age- and sex-matched group of healthy controls, as previously described [11].

Written informed consent was obtained from all participants and/or their legal guardians prior to study inclusion. The study protocol was approved by the institutional review board of Hannover Medical School (approval number 504–2009) and conducted in accordance with the Declaration of Helsinki.

### Anthropometrics and blood pressure measurement

Anthropometric measurements including body weight and height were obtained at the time of study inclusion.

Blood pressure measurements were performed using a validated oscillometric device (Dinamap V100, GE Healthcare, Chicago, IL) following a standardized measurement protocol. Systolic and diastolic blood pressure *z*-scores were calculated based on reference data from the National High Blood Pressure Education Program Working Group on High Blood Pressure in Children and Adolescents [21].

### Echocardiography

All kidney transplant recipients underwent comprehensive transthoracic echocardiography with a median interval of 7 days (range, 0–56 days) between echocardiographic examination and CMR imaging. All examinations were performed by a single investigator with expertise in pediatric echocardiography, following a standardized protocol in accordance with the guidelines of the Pediatric Council of the American Society of Echocardiography [22].

Echocardiographic studies were conducted using a Philips CX50 ultrasound system (Philips Medical Systems, Bothell, WA) equipped with a 5-MHz transducer. Left ventricular (LV) end-diastolic wall thickness and LV end-diastolic dimensions were obtained from the parasternal short-axis view at the level of the papillary muscles using M-mode imaging. Left ventricular mass index (LVMI) was calculated as proposed by Chinali et al. [23]. Left ventricular ejection fraction (EF) was assessed using the biplane modified Simpson method.

Diastolic function was evaluated using pulsed-wave Doppler and tissue Doppler imaging. Transmitral inflow velocities were recorded in the apical four-chamber view with the sample volume positioned between the tips of the mitral valve leaflets optimally aligned with blood flow. Early (E) and late (A) diastolic transmitral flow velocities were measured, and the E/A ratio was calculated. Tissue Doppler imaging was used to obtain peak early (e′) and late (a′) diastolic velocities at both the septal and lateral mitral annulus. The E/e′ ratio was calculated accordingly.

Isovolumic relaxation time (IVRT) was derived from tissue Doppler recordings and measured from the end of the systolic (S) wave to the onset of the subsequent e′ wave. Pulmonary venous flow was assessed in the apical four-chamber view with the pulsed-wave Doppler sample volume positioned as distally as possible within the right upper pulmonary vein. Measurements included peak systolic velocity (PVFsys), peak diastolic velocity (PVFdia), and atrial reversal velocity (PVF-AR).

Age-specific reference values published by Eidem et al. were used for the evaluation of diastolic parameters [24]. The prevalence of diastolic dysfunction was evaluated using the parameters E/A ratio, septal E/e′, lateral E/e′, septal IVRT, PVFsys, and PVFdia [25, 26]. All echocardiographic parameters were measured five times, and the median value was used for subsequent analyses. Measurements were included only when image quality was deemed excellent and unambiguous; consequently, not all parameters were available for all participants.

### CMR imaging

CMR imaging was performed as previously described in detail for this cohort [11]. All examinations were conducted using a standardized imaging protocol and quality criteria. Native T1 mapping was employed to assess diffuse myocardial alterations, serving as a surrogate marker of myocardial fibrosis.

In addition to septal native T1 assessment, lateral myocardial native T1 values were obtained to enable a more comprehensive evaluation of regional myocardial involvement. Lateral native T1 was measured using manually contoured, curved region of interest (ROI) placed along the lateral wall of the left ventricle, carefully avoiding inclusion of the blood pool and epicardial fat. The placement of septal and lateral ROIs is illustrated in the online resource (Fig. S1). The same imaging parameters, acquisition sequences, and quality criteria applied for septal native T1 measurements were used for lateral native T1 assessment to ensure comparability.

All CMR analyses were performed in a blinded fashion to reduce an observational bias, and measurements were included only when image quality met predefined standards.

### Statistical analysis

Continuous variables are presented as mean ± standard deviation (SD), and categorical variables as absolute numbers and percentages. Data distribution was assessed using the Shapiro–Wilk test. Comparisons of continuous variables between KTx recipients and healthy controls were performed using unpaired *t* tests, whereas categorical variables were compared using the chi-squared test.

Associations between echocardiographic parameters and native T1 values were evaluated using multivariable linear regression models adjusted for age, sex, and height. Receiver operating characteristics (ROC) curves were generated for septal E/e′ to assess its diagnostic performance for detecting an elevated septal and lateral native T1. The area under the curve (AUC) was calculated, and the optimal cut-off was derived using positive likelihood ratios (LR +) to assess how strongly an elevated E/e′ increases the probability of an elevated septal and lateral native T1.

Statistical significance was defined as a two-sided *p* value < 0.05. All statistical analyses were performed using SAS Enterprise Guide version 7.1 (SAS Institute, Cary, NC).

## Results

### Characteristics of the study population

We included 46 children, adolescents, and young adults following pediatric KTx in our study (Table 1). Mean age at study inclusion was 16 ± 4 years, and 22 participants (48%) were female. Mean time since KTx was 7.9 ± 5.3 years. Twenty KTx recipients (43%) had undergone preemptive KTx without prior dialysis.

**Table 1:** Characteristics of the 46 patients after pediatric KTx. Data are either expressed as absolute numbers and percentage in brackets or as mean ± standard deviation, with the range in brackets

**Variable**	**Number (percentage)**	**Mean ± SD (range)**
**Demographics**		
**Age [years]**		16.0 ± 3.5 (7–25)
**Sex, male/female**	24 (52)/22 (48)	
**Underlying disease**		
CAKUT	27 (59)	
Non-CAKUT	19 (41)	
**Anthropometrics and blood pressure**		
**Height**		
Absolute [cm]		160.3 ± 15.3 (115.5–184.5)
***z***-score		− 0.4 ± 1.3 (− 2.8–3.2)
**Weight**		
Absolute [kg] ***z***-score		55.4 ± 15.1 (20.3–99.6) − 0.1 ± 1.2 (− 2.9–2.2)
**SBP**		
Absolute [mmHg]		120.7 ± 14.5 (97–155)
***z***-score		0.9 ± 1.4 (− 1.9–4.3)
**DBP**		
Absolute [mmHg]		70.9 ± 11.3 (52–107)
***z***-score		0.5 ± 1.1 (− 1.2–3.5)
**RHR**		
Absolute [1/min]		79.8 ± 12.3 (54.0–108.0)
***z***-score		0.4 ± 1.0 (− 1.7–2.5)
**Transplantation details**		
**Age at transplantation [years]**		8.1 ± 4.8 (1–16.9)
**Time since transplantation [years]**		7.9 ± 5.3 (0.7–23.6)
**Re-transplantation**	3 (7)	
**Dialysis details**		
**No dialysis before transplantation**	20 (43)	
**Time on dialysis [months]**^**a**^		
HD/PD		16.0 ± 11.3 (1.2–42.5)
HD		15.8 ± 15.9 (0.4–42.5)
PD		15.3 ± 9.0 (1.2–33.5)
**Immunosuppressive medication**		
Calcineurin inhibitor	42 (91)	
mTOR inhibitor	37 (80)	
Mycophenolate-mofetil	11 (24)	
Glucocorticosteroid	24 (52)	
**Kidney function**		
**Estimated GFR [ml/min/1.73 m**^**2**^**]**		60.5 ± 32 (16–144.5)

*CAKUT* congenital anomalies of kidney and urinary tract, *CKD* chronic kidney disease, *DBP* diastolic blood pressure, *GFR* glomerular filtration rate, *HD* hemodialysis, *MR* mineralocorticoid receptor, *PD* peritoneal dialysis, *RHR* resting heart rate, *SBP* systolic blood pressure

^a^one patient received HD after PD

Congenital anomalies of the kidney and urinary tract (CAKUT) represented the most common underlying diagnosis, accounting for 59% of transplantations. Among participants who required dialysis prior to transplantation, the cumulative duration of dialysis was 16.0 ± 11.3 months. Mean eGFR at study inclusion was 60.5 ± 32.0 ml/min/1.73 m^2^.

Most KTx recipients were receiving calcineurin inhibitor–based immunosuppressive therapy (*n* = 42; 91%), frequently in combination with mechanistic target of rapamycin (mTOR) inhibitors (*n* = 37; 80%) or mycophenolate mofetil (*n* = 11; 24%). Steroid therapy was administered in 24 participants (52%).

### CMR imaging: assessment of septal and lateral structural myocardial alterations

As previously reported for this cohort, septal native T1 values were significantly higher in KTx recipients compared with age- and sex-matched healthy controls [11].

To further assess regional myocardial involvement, native T1 values were additionally analyzed in the lateral wall of the left ventricle. Mean lateral native T1 was significantly higher in KTx recipients than in healthy controls (1140.5 ± 56.5 ms vs. 1108.2 ± 39.9 ms; *p* = 0.003; Fig. S2). Elevated lateral native T1 values, defined as values exceeding 1184 ms, were observed in 8 of 44 kidney transplant recipients (18%). Seven of the eight participants with elevated lateral native T1 values also exhibited elevated septal native T1 values.

Other CMR-derived parameters, including volumetric and functional measurements, as well as their comparison with echocardiographic findings, are provided in the online resource (Table S1).

### Echocardiographic findings

Echocardiographic measurements are summarized in the online resource in Table S2. Mean LVMI was 39.2 ± 8.1 g/m^2.16^, and left ventricular hypertrophy was present in nine participants (20%). Left ventricular systolic function was preserved in all KTx recipients, with a mean EF of 65.9 ± 2.6%.

**Fig. 1:**
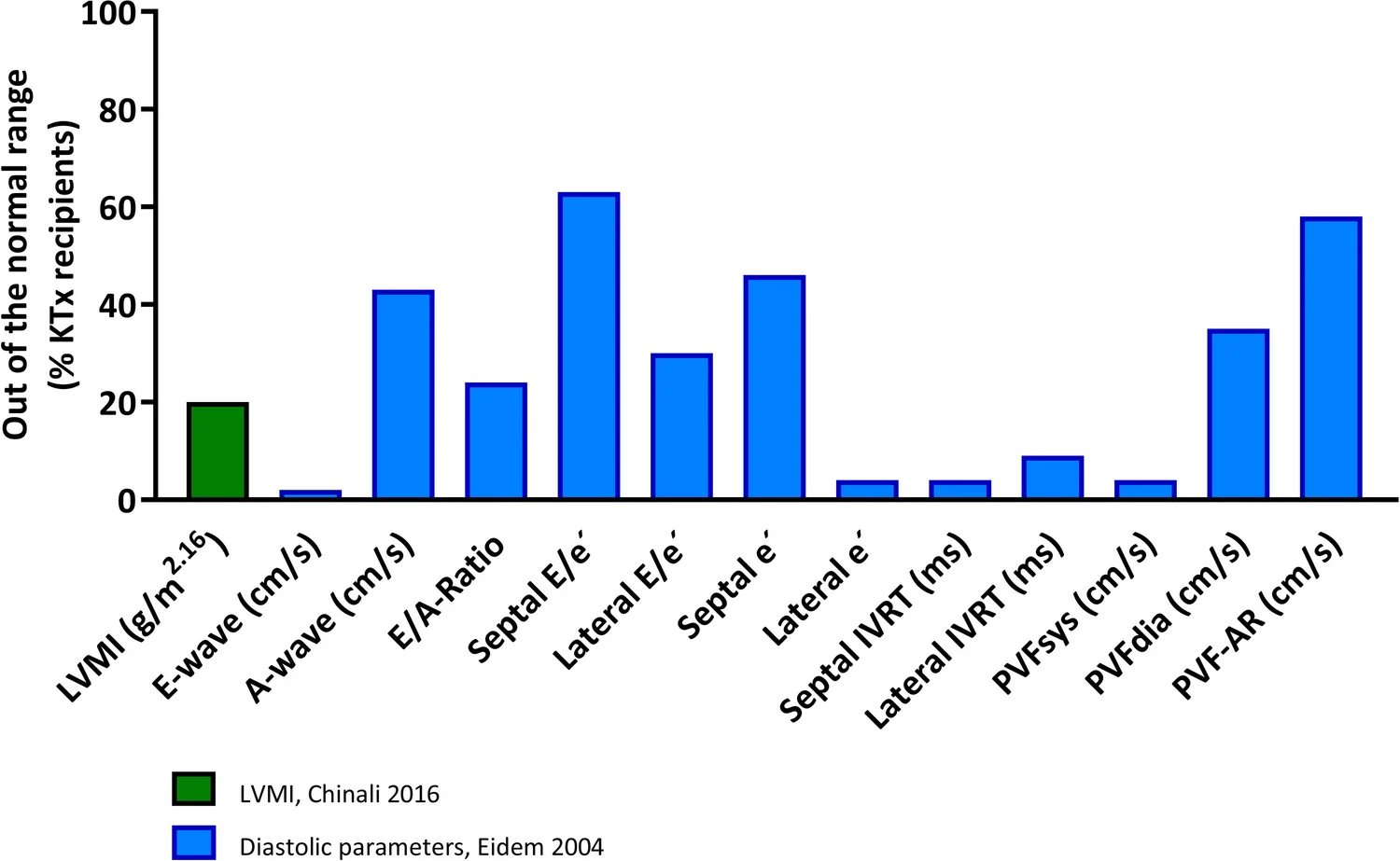
Echocardiographic parameters out of the age-specific normal range. Different echocardiographic parameters were compared with values from reference studies, and the proportion of measurements being outside the respective normal ranges is reported. IVRT, isovolumetric relaxation time; LVMI, left ventricular mass index; PVF-AR, pulmonary venous flow-atrial reversal; PVFdia, diastolic pulmonary venous flow; PVFsys, systolic pulmonary venous flow

Diastolic echocardiographic parameters frequently exhibited values outside the age-specific reference ranges (Fig. 1). According to the reference values published by Eidem et al. [24], 40 KTx recipients (87%) exhibited at least one of these relevant diastolic parameters outside the normal range, and more than half of the cohort (*n* = 24; 52%) showed abnormalities in two or more parameters. Focusing on a key parameter of left ventricular diastolic function, the septal E/e′ ratio, the prevalence of abnormal values was high, with 29 participants (63%) exceeding the age-specific normal range. Peak diastolic pulmonary venous flow velocity was increased in 16 of 46 participants (35%), and atrial reversal velocity (PVF-AR) was increased in 18 of 31 participants (58%) with available measurements.

Abnormal diastolic parameters were more frequently observed at the interventricular septum than at the lateral mitral annulus. Specifically, reduced septal e′ velocities were present in 46% of participants compared with 4% at the lateral annulus, and elevated septal E/e′ ratios were observed in 63% compared with 30% at the lateral annulus.

### Association of echocardiographic parameters reflecting diastolic function and native T1

**Fig. 2:**
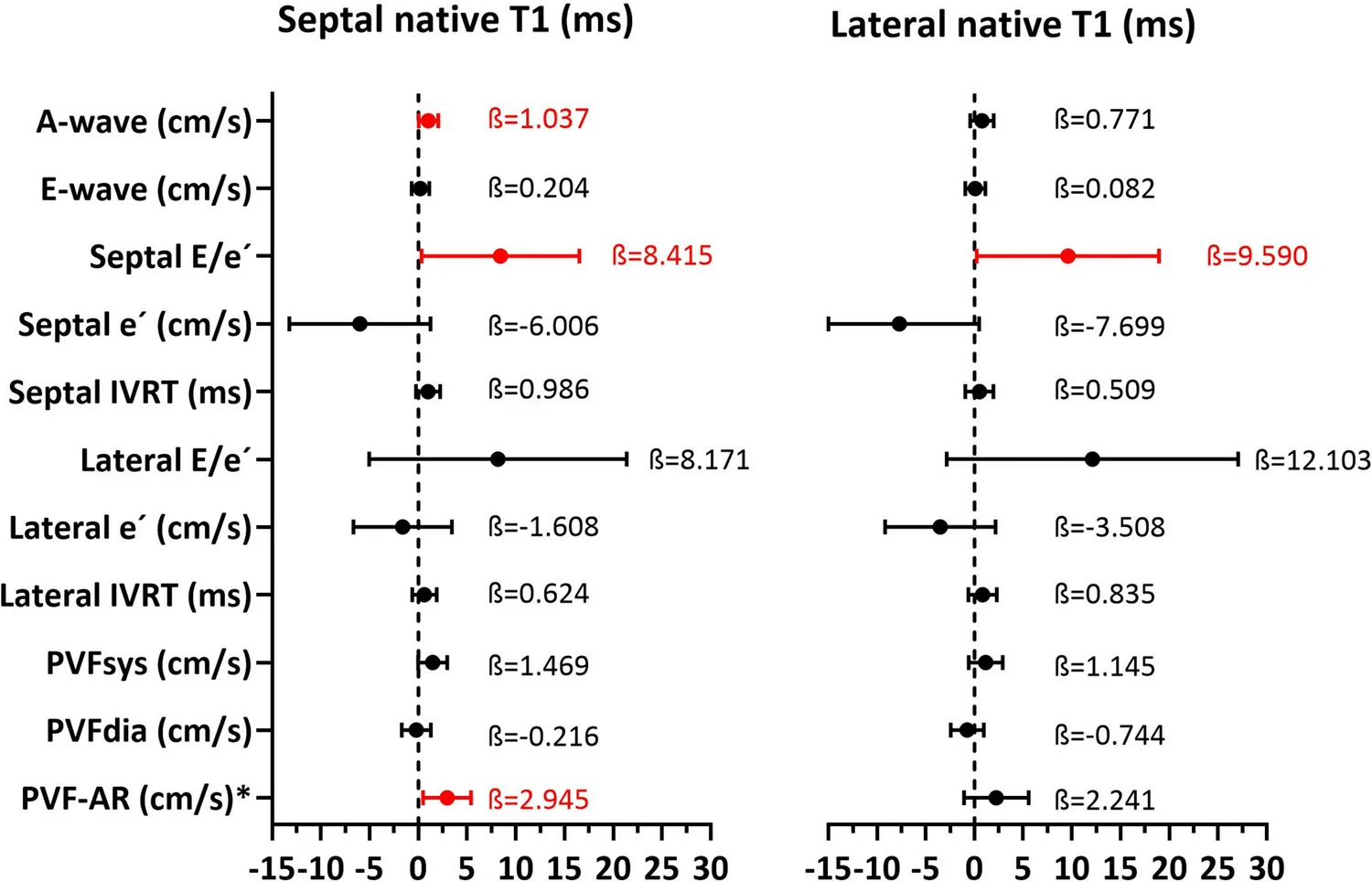
Association of echocardiographic markers of diastolic function and septal and lateral native T1. Each parameter was tested for its influence on native T1 independent of age, sex, and height. Reported are the standardized coefficient (β) and the 95% confidence interval. Significant associations are highlighted in red. IVRT, isovolumetric relaxation time; PVF-AR, pulmonary venous flow-atrial reversal; PVFdia, diastolic pulmonary venous flow; PVFsys, systolic pulmonary venous flow; T1, T1 time; β, standardized coefficient. *For PVF-AR *n* = 31 measurements were available for association with septal native T1 and *n* = 30 measurements for association with lateral native T1

Figure 2 illustrates associations between echocardiographic parameters reflecting diastolic function and native T1 values. Multivariable linear regression analyses adjusted for age, sex, and height demonstrated significant associations between septal native T1 and septal E/e′ ratio (β = 8.415; *p* = 0.042), transmitral A-wave velocity (β = 1.037; *p* = 0.045), and pulmonary venous flow atrial reversal velocity (β = 2.945; *p* = 0.02). For lateral native T1, septal E/e′ ratio was the only echocardiographic parameter showing a significant association in multivariable analyses (β = 9.590; *p* = 0.045).

**Fig. 3:**
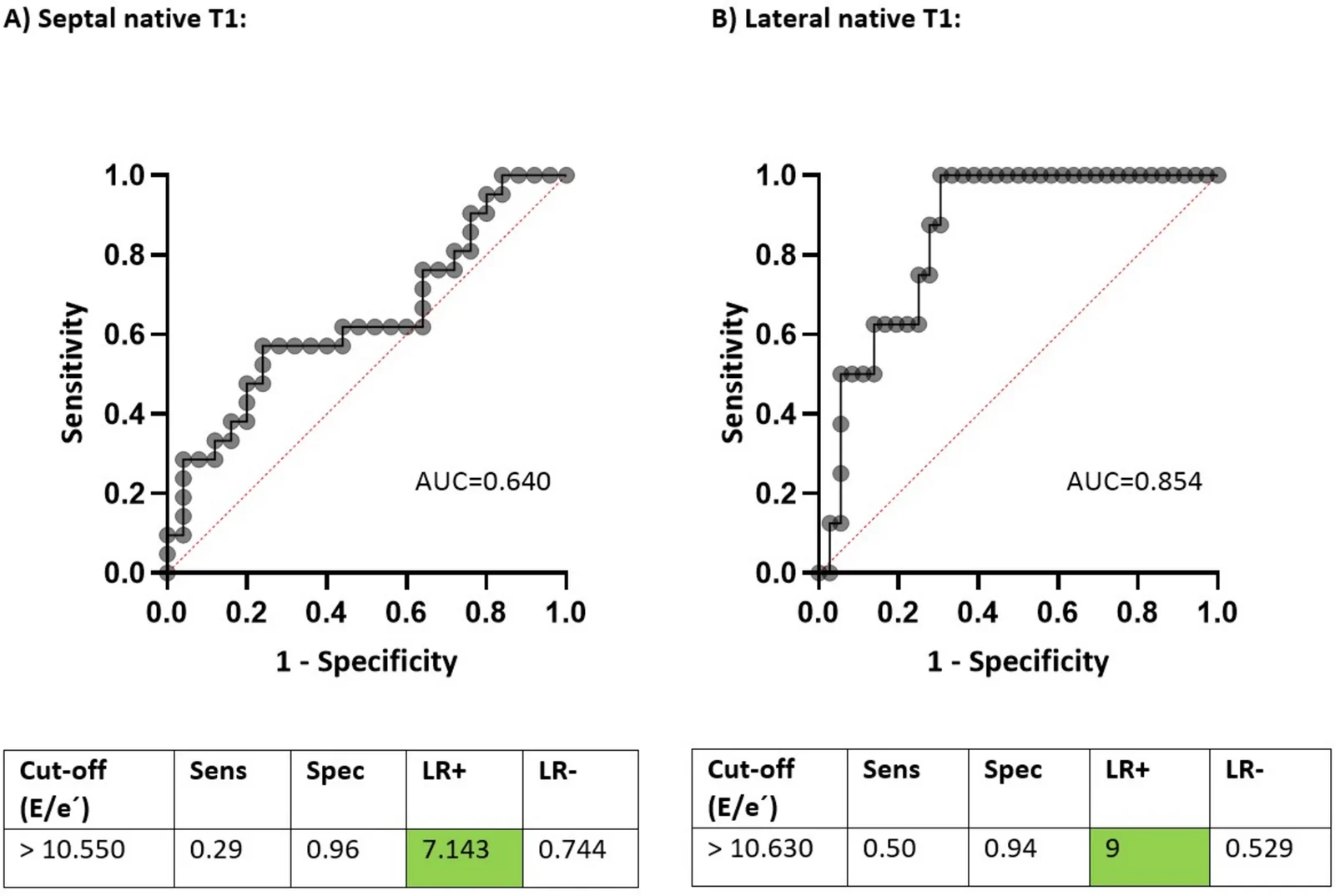
ROC curves for septal E/e′ to identify an elevated septal (**A**) and lateral (**B**) native T1. The area under the curve (AUC) was calculated for septal and lateral native T1. Cut-off analysis was performed by selecting the septal E/e′ value with the highest positive likelihood ratio (LR +) (highlighted in green). AUC, area under the curve; LR +, positive likelihood ratio; LR-, negative likelihood ratio; ROC, receiver operating characteristics; Sens, sensitivity; Spec, specificity; T1, T1 time

All seven KTx recipients with an elevated septal and lateral native T1 exhibited septal E/e′ ratios outside the age-specific normal range. We performed ROC curve analyses to evaluate the diagnostic performance of septal E/e′ for detecting KTx recipients with an elevated septal and lateral native T1 (Fig. 3). For septal native T1, septal E/e′ showed a modest AUC of 0.640, whereas for lateral native T1, the AUC was higher at 0.854. Using positive likelihood ratios (LR +), the optimal cut-offs of septal E/e′ were 10.550 for detecting an elevated septal native T1 (LR +  = 7.143) and 10.630 for detecting an elevated lateral native T1 (LR +  = 9). Based on the reference values published by Eidem et al. [25], septal E/e′ was elevated in 29 (63%) of our KTx recipients. When applying the calculated cut-off value of 10.550 for septal E/e′, only seven KTx recipients (15%) remained with a high likelihood of exhibiting an elevated septal native T1 on CMR imaging. Similarly, applying the corresponding cut-off values for detecting an elevated lateral native T1 identified only six KTx recipients (14%) with a high likelihood of structural myocardial alterations.

## Discussion

In this cross-sectional study of children, adolescents, and young adults after pediatric KTx, we demonstrate a high prevalence of diastolic echocardiographic abnormalities despite preserved systolic left ventricular function. Structural myocardial alterations assessed by native T1 mapping CMR imaging were present in a substantial proportion of patients, extending previous observations in this cohort [11]. Importantly, specific diastolic echocardiographic parameters were associated with native T1 values, linking functional abnormalities detected by routine echocardiography to underlying structural myocardial changes. Together, these findings indicate that pediatric KTx recipients with myocardial alterations on CMR imaging already exhibit detectable abnormalities in diastolic echocardiographic parameters, suggesting that echocardiography may serve as a pragmatic first-line approach to identify patients at increased risk for myocardial involvement and to guide targeted use of advanced imaging.

The predominance of diastolic echocardiographic abnormalities observed in this cohort is consistent with previous studies in children with CKD and after KTx, which have consistently reported functional alterations affecting diastolic rather than systolic parameters [13–15]. In line with these reports, left ventricular systolic function was preserved in all participants, whereas deviations from age-specific reference values were frequently observed for diastolic indices. Notably, diastolic abnormalities occurred largely independent of left ventricular hypertrophy, supporting the concept that functional myocardial involvement may precede overt structural remodeling detectable by conventional echocardiographic measures [15, 16]. Together, these findings reinforce the relevance of diastolic echocardiographic assessment as a sensitive marker of early cardiac involvement in pediatric KTx recipients.

A central finding of this study is the association between diastolic echocardiographic parameters and structural myocardial alterations assessed by native T1 mapping on CMR imaging. These results suggest that functional abnormalities detected by routine echocardiography may coincide with underlying myocardial changes in pediatric KTx recipients. In particular, the septal E/e′ ratio, which reflects impaired myocardial relaxation and elevated left ventricular filling pressures, was associated with both septal and lateral native T1 values [19, 25]. In addition, transmitral A-wave velocity and pulmonary venous flow atrial reversal were associated with septal native T1, consistent with altered ventricular compliance and increased atrial contribution to left ventricular filling [26]. While these associations do not imply causality, they identify echocardiographic parameters that are linked to structural myocardial alterations detected by CMR imaging in this patient population.

Diastolic echocardiographic abnormalities were more pronounced at the interventricular septum than at the lateral mitral annulus, particularly for e′ velocity and E/e′ ratio. Previous echocardiographic studies have suggested that septal diastolic parameters may provide a more sensitive reflection of impaired myocardial relaxation and filling characteristics than lateral measurements, especially in the presence of advanced myocardial remodeling [27]. Concordantly, regional differences in myocardial native T1 values between the interventricular septum and non-septal myocardial regions are well recognized, with higher septal native T1 values reported in both healthy individuals and patients with kidney disease [28, 29]. In cohorts of patients on hemodialysis, septal native T1 has been shown to exceed non-septal values more consistently than in healthy controls, suggesting a particular susceptibility of the interventricular septum in the context of uremic cardiomyopathy [30, 31]. In line with these observations, in this study, elevations in lateral native T1 rarely occurred without concomitant septal native T1 elevation. A possible interpretation is that structural myocardial alterations initially involve the interventricular septum and subsequently extend to the lateral wall. Based on positive likelihood ratios, a septal E/e′ > 10.630 was associated with a nine-fold increase in the probability of an elevated lateral native T1. Application of this cut-off value identified a small subgroup of six KTx recipients as high-risk patients for exhibiting structural myocardial changes. These findings suggest that markedly elevated septal E/e′ may be predictive of advanced structural myocardial alterations and could support the selective use of CMR imaging for further characterization.

The present findings have relevant clinical implications for cardiovascular surveillance in pediatric KTx recipients. Routine echocardiography is widely available and already integrated into post-transplant follow-up, whereas CMR imaging remains resource-intensive and not universally applicable. The observed associations between diastolic echocardiographic abnormalities, especially septal E/e′, and native T1 values suggest that echocardiography may help identify patients at increased risk for underlying myocardial alterations who could benefit from further structural assessment. Diffuse myocardial fibrosis has been associated with adverse cardiovascular outcomes, including heart failure and arrhythmias, in adult patients with advanced kidney disease [7, 32]. In addition, elevated global native T1 values have been linked to increased mortality risk in patients on hemodialysis [8], underscoring the clinical relevance of early detection of myocardial involvement. Although comparable outcome data are currently lacking in pediatric KTx populations, the constellation of preserved systolic function, frequent diastolic abnormalities, and structural myocardial alterations observed in the present study may be viewed as compatible with early features of heart failure with preserved ejection fraction (HFpEF), an entity well described in adults but not yet clearly defined in pediatric populations [33, 34]. In this context, a risk-adapted approach integrating echocardiographic assessment of diastolic function may support more targeted use of CMR imaging, rather than universal advanced imaging. Such a strategy could improve the identification of subclinical myocardial involvement while optimizing resource utilization and minimizing patient burden, particularly in pediatric settings. Our initial CMR analyses in KTx recipients identified an association between structural cardiac damage and blood pressure control [11], highlighting the potential clinical relevance of optimized antihypertensive management after KTx. Nevertheless, optimal blood pressure management strategies and target blood pressure values in this setting remain insufficiently defined. The ongoing SOPHOCLES study was initiated to address whether optimized blood pressure control can favorably influence structural and functional cardiac alterations [35].

This study has several limitations that warrant consideration. The cross-sectional design limits interpretation of temporal relationships and does not allow conclusions regarding the progression or prognostic relevance of the observed findings. Accordingly, the reported associations between diastolic echocardiographic parameters and native T1 values should be interpreted as descriptive rather than causal. The sample size was modest and derived from a single center, which may limit generalizability. To further investigate the potential effects of medication, particularly immunosuppressive regimens, on the observed echocardiographic and CMR abnormalities, additional studies with larger sample sizes are required. Multivariable regression analyses were exploratory and aimed at identifying potential associations rather than establishing definitive predictors. In addition, pulmonary venous flow parameters could not be obtained in all participants, which reduced the number of observations available for selected analyses. Finally, differences between echocardiographic and CMR-derived measurements should be interpreted in the context of known methodological variability between imaging modalities.

## Conclusion

In summary, diastolic echocardiographic abnormalities are common in children and young adults after pediatric KTx and are associated with structural myocardial alterations assessed by native T1 mapping on CMR imaging. Routine echocardiographic assessment of diastolic function, particularly the identification of markedly elevated septal E/e′ values, may help identify a high-risk subgroup of pediatric KTx recipients with an increased likelihood for myocardial involvement and support a risk-adapted, targeted use of advanced imaging in long-term post-transplant follow-up. Further longitudinal studies are needed to validate these findings and to clarify their clinical and prognostic relevance.

## Supplementary Information

Below is the link to the electronic supplementary material.

Graphical abstract (PPTX 944 kb)

Supplementary file1 (PDF 291 kb)

## Data Availability

The datasets generated and analyzed during the current study are available from the corresponding author on reasonable request**.**
