# Supplementary material for: Diastolic echocardiographic parameters identify pediatric kidney transplant recipients with structural myocardial alterations
Source: Pediatr Nephrol. 2026 Jun 27;41(11):4165–75. doi: 10.1007/s00467-026-07416-1 (PMC13612606; doi:10.1007/s00467-026-07416-1)
Supplement: Supplementary file 1 [file 467_2026_7416_MOESM2_ESM.pdf]

Supplementary Material to:

Diastolic Echocardiographic Parameters Identify Pediatric Kidney Transplant Recipients With Structural Myocardial Alterations

Jeannine von der Born MD[1,2*]; Tim Alexander Ubenauf[1,3*]; Rizky I. Sugianto DDS, PhD[1]; Carl Grabitz MD[1]; Elena Lehmann MSc[1]; Nima Memaran MD[1]; Nigar Babazade[4]; Samir Sarikouch MD, PhD[5]; Diane Miriam Renz MD[4]; Bernhard Magnus Wilhelm Schmidt MD, MSc[6]; Anette Melk MD, PhD[1]

[1] Department of Pediatric Kidney- and Liver Diseases, Metabolics and Neuropediatrics, Hannover Medical School, Hannover, Carl-Neuberg-Str. 1, 30625 Hannover, Germany.

[2] AVIATOR – Advanced Clinician Scientist Program, Dean's Office for Academic Career Development, Hannover Medical School, Carl-Neuberg-Str. 1, 30625 Hannover, Germany.

[3] KlinStrucMed Program, Dean's Office for Academic Career Development, Hannover Medical School, Carl-Neuberg-Str. 1, 30625 Hannover, Germany.

[4] Institute of Diagnostic and Interventional Radiology, Hannover Medical School, Hannover, Carl-Neuberg-Str. 1, 30625 Hannover, Germany.

[5] Department of Cardiothoracic, Transplantation and Vascular Surgery, Hannover, Carl-Neuberg-Str. 1, 30625 Hannover, Germany.

[6] Department of Nephrology and Hypertension, Hannover Medical School, Hannover, Carl-Neuberg-Str. 1, 30625 Hannover, Germany.

*J. von der Born and T. A. Ubenauf contributed equally

Corresponding author: Anette Melk MD, PhD

Department of Pediatric Kidney- and Liver Diseases, Metabolics and Neuropediatrics, Hannover Medical School; Carl-Neuberg-Str. 1; 30625 Hannover; Germany

Email: melk.anette@mh-hannover.de

ORCID: 0000-0002-8164-6318

Table S1: Comparison of LVM, LVMI and EF measurements between CMR imaging and echocardiography. Data are presented as mean with standard deviation and range in brackets.

Variable	Echocardiography	CMR Imaging
**LVM [g]**	113.8±32.7 (52.5-200.7)	97.5±28.1 (44.0–177.0)
**LVMI [g/m²]**		61.9±14.2 (39.0–104.0)
**LVMI [g/m$^{2.16}$]***	39.2 ± 8.1 (25.4-59.0)	
**EF [%]**	65.9 ± 2.6 (61.0-70.6)	67.3±3.8 (59.0–76.0)

Abbreviations: CMR, cardiac magnetic resonance; EF, ejection fraction; LVM, left ventricular mass; *LVMI, left ventricular mass index according to Chinali et al.[1].

[1]Chinali M, Emma F, Esposito C, et al. Left Ventricular Mass Indexing in Infants, Children, and Adolescents: A Simplified Approach for the Identification of Left Ventricular Hypertrophy in Clinical Practice. J Pediatr. Mar 2016;170:193-8. doi:10.1016/j.jpeds.2015.10.085

Table S2: Echocardiographic measurements and their percentage being out of norm. Data are expressed as mean ± standard deviation and with their range.

Variable	Mean ± SD	Range
**Diastolic Function**		
**E-wave [cm/s]**	105.5 ± 17.3	56.4 -147.0
**A-wave [cm/s]**	61.6 ± 14.1	36.6 - 115.0
**E/A-Ratio**	1.8 ± 0.4	1.2 - 3.1
**Septal E/e'**	8.7 ± 1.8	5.2 - 13.9
**Lateral mitral E/e'**	5.8 ± 1.1	3.1 - 8.4
**Septal e' [cm/s]**	12.4 ± 2.0	7.8 - 17.1
**Lateral mitral e' [cm/s]**	18.4 ± 3.0	11.0 - 24.7
**Septal IVRT [ms]**	70.0 ± 11.9	49.0 - 99.0
**Lateral IVRT [ms]**	63.2 ± 12.1	45.0 - 92.0
**PVFsys [cm/s]**	57.1 ± 9.7	38.5 - 79.8
**PVFdia [cm/s]**	67.3 ± 10.2	30.7 - 83.5
**PVF-AR [cm/s] (n=31)**	29.8 ± 7.5	19.7 - 48.2
**Systolic function**		
**EF [%]**	65.9 ± 2.6	61.0 - 70.6
**Structural measures**		
**LVMI [$g/m^{2.16}$]**	39.2 ± 8.1	25.4 - 59.0

Abbreviations: EF, ejection fraction**;** LVMI, left ventricular mass index according to Chinali et al.[1]; IVRT, isovolumetric relaxation time; PVF-AR, pulmonary venous flow - atrial reversal; PVFdia, diastolic pulmonary venous flow; PVFsys, systolic pulmonary venous flow; SD, standard deviation.

[1]Chinali M, Emma F, Esposito C, et al. Left Ventricular Mass Indexing in Infants, Children, and Adolescents: A Simplified Approach for the Identification of Left Ventricular Hypertrophy in Clinical Practice. J Pediatr. Mar 2016;170:193-8. doi:10.1016/j.jpeds.2015.10.085

Fig. S1 Basal short axis slices of T1 mapping with manually placed septal (pink) and blue (lateral) region of interest (ROI). ROIs were manually contoured to avoid tissue interfaces between myocardium and blood pool/ epicardial fat.

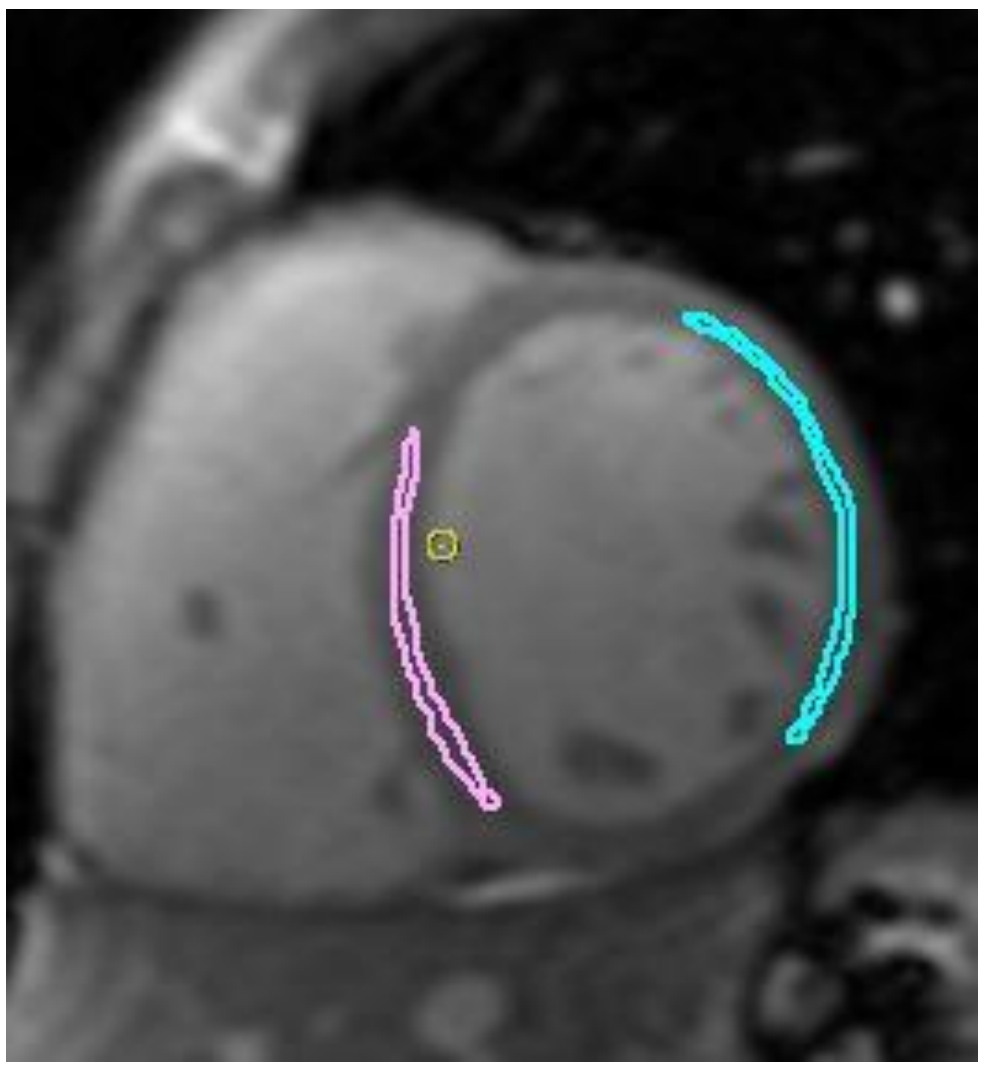

Fig. S2 Lateral native T1 in KTx recipients and controls. Native T1 time presented as absolute values, bars indicate the mean.

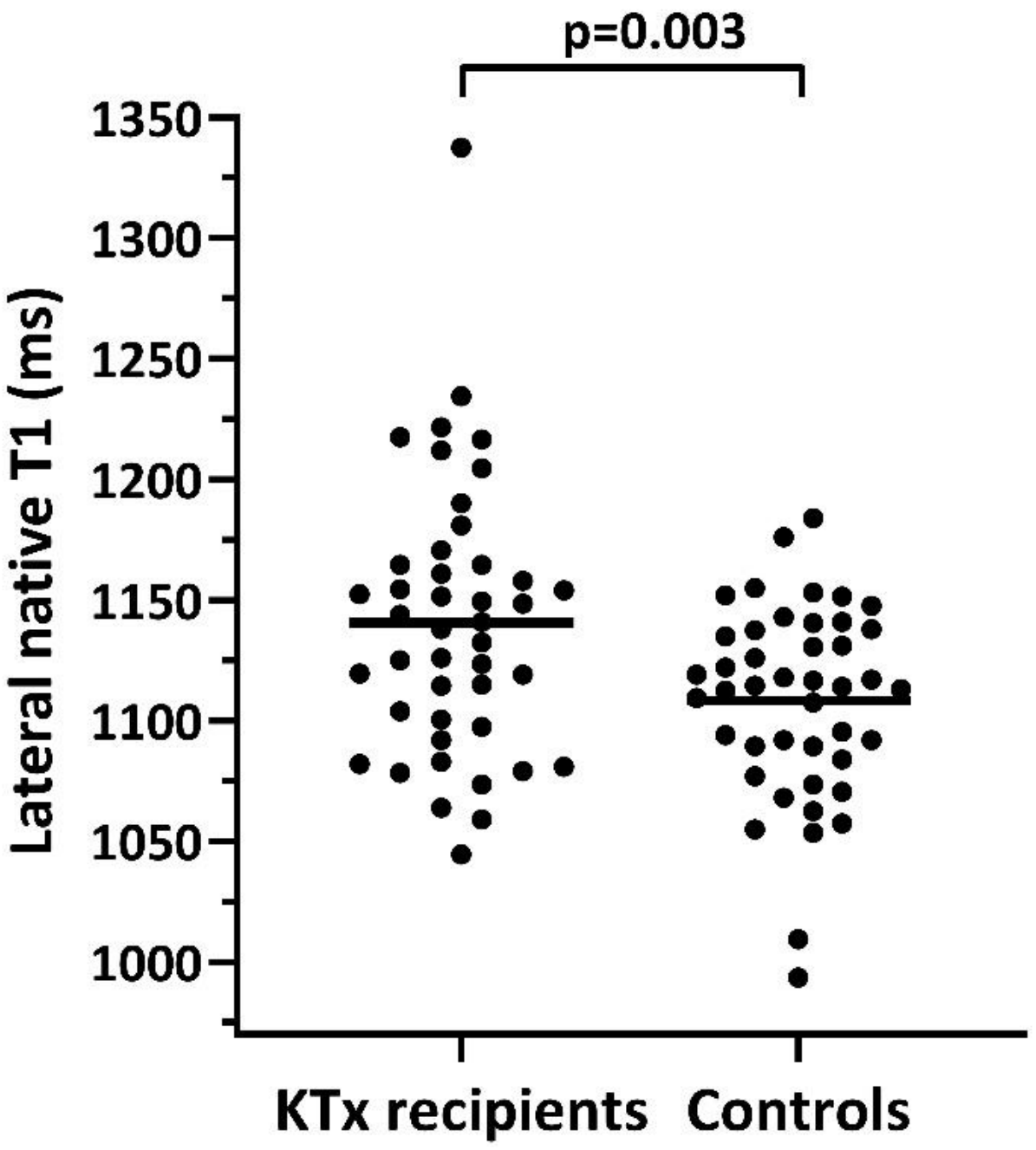

Abbreviations: KTx, kidney transplantation; T1, T1 time
